# Screening of GABA-Producing Lactic Acid Bacteria from Thai Fermented Foods and Probiotic Potential of *Levilactobacillus brevis* F064A for GABA-Fermented Mulberry Juice Production

**DOI:** 10.3390/microorganisms9010033

**Published:** 2020-12-24

**Authors:** Jirapat Kanklai, Tasneem Chemama Somwong, Patthanasak Rungsirivanich, Narumol Thongwai

**Affiliations:** 1 Department of Biology, Faculty of Science, Chiang Mai University, Chiang Mai 50200, Thailand; jirapat18101987@gmail.com (J.K.); patthanasak_bas@hotmail.com (P.R.); 2 Graduate School, Chiang Mai University, Chiang Mai 50200, Thailand; 3 Department of Biology, Faculty of Science and Technology, Princess of Naradhiwas University, Naradhiwas 96000, Thailand; Tasneem.s@pnu.ac.th; 4 Research Center in Bioresources for Agriculture, Industry and Medicine, Chiang Mai University, Chiang Mai 50200, Thailand

**Keywords:** antioxidant, antibacterial, GABA, glutamate, *Levilactobacillus*

## Abstract

Gamma-aminobutyric acid (GABA), the inhibitory neurotransmitter, can be naturally synthesized by a group of lactic acid bacteria (LAB) which is commonly found in rich carbohydrate materials such as fruits and fermented foods. Thirty-six isolates of GABA-producing LAB were obtained from Thai fermented foods. Among these, *Levilactobacillus brevis* F064A isolated from Thai fermented sausage displayed high GABA content, 2.85 ± 0.10 mg/mL and could tolerate acidic pH and bile salts indicating a promising probiotic. Mulberry (*Morus* sp.) is widely grown in Thailand. Many mulberry fruits are left to deteriorate during the high season. To increase its value, mulberry juice was prepared and added to monosodium glutamate (MSG), 2% (*w/v*) prior to inoculation with 5% (*v/v*) of *L. brevis* F064A and incubated at 37 °C for 48 h to obtain the GABA-fermented mulberry juice (GABA-FMJ). The GABA-FMJ obtained had 3.31 ± 0.06 mg/mL of GABA content, 5.58 ± 0.52 mg gallic acid equivalent/mL of antioxidant activity, 234.68 ± 15.53 mg cyanidin-3-glucoside/mL of anthocyanin, an ability to inhibit growth of *Bacillus cereus* TISTR 687, *Salmonella* Typhi DMST 22842 and *Shigella dysenteriae* DMST 1511, and 10.54 ± 0.5 log_10_ colony-forming units (CFU)/mL of viable *L. brevis* F064A cell count. This GABA-FMJ was considered as a potential naturally functional food for human of all ages.

## 1. Introduction

Nowadays people are interested in high-quality functional healthy foods which contain valuable bioactive compounds and/or beneficial microorganisms that can promote health. Lactic acid bacteria (LAB), a prominent probiotic, are generally used in food, agro- and pharmaceutical industries due to their “Generally Recognized as Safe” (GRAS) property [1]. Besides lactic acid, various organic acids and bioactive compounds are produced by LAB i.e., bacteriocins and γ-aminobutyric acid (GABA).

GABA functions as an inhibitory neurotransmitter in the human brain [2]. Normally, exogenous GABA is believed to be unable to cross the blood brain barrier (BBB), however, some recent research has shown evidence that GABA may pass the BBB or the exogenous GABA may induce the production of endogenous GABA [3]. GABA produced by gut microbiota can affect the central nervous system (CNS) via the enteric nervous system (ENS) and modulate the gut–brain axis [4]. Moreover, GABA has activities as antidepressant [5], anti-diabetic [6,7], anti-hypertension [8], neuroprotecting agent [9], cardiovascular regulation agent [10], lung adenocarcinoma suppressor [11] and rat plasma growth hormone enhancer [12]. The biosynthesis of GABA in LAB is responsible by an enzyme glutamate decarboxylase (GAD) using L-glutamate as a substrate. GAD system is a member of an amino acid-dependent acid resistance (AR) system which is used to maintain the intracellular pH homeostasis through GABA biosynthesis [13].

A fermented beverage (FB) is non-dairy fermented product which is produced by yeast and/or lactic acid bacteria fermentation from different sources such as whey, grain, vegetables or fruit juice. FB can improve nutrition components and become a health-promoting product [14]. In Thailand, mulberry (*Morus* spp.) is widely planted but the mulberry fruit market value is quite low. Due to its richness in beneficial bioactive compounds and medicinal functions such as the properties of prevention and treatment of a sore throat, obesity, diabetes mellitus and hypertension [15], investigations to extract the useful compounds as well as to develop healthy food products will add more value to the mulberry fruits and benefit not only gardeners but also customers of all ages. Hence, this study aimed to isolate and screen for effective GABA-producing LAB, to elucidate the human probiotic potential of the selected LAB strain, to investigate mulberry juice properties, to create a product of GABA-fermented mulberry juice (GABA-FMJ) and to analyze the antioxidant and antibacterial properties of the product obtained. The data acquired will provide information about an alternative healthy beverage which could be consumed for prevention and protection of some symptoms and/or diseases both infectious and non-infectious.

## 2. Materials and Methods 

### 2.1. Isolation and Screening of γ-Aminobutyric Acid (GABA)-Producing Lactic Acid Bacteria (LAB)

De Man, Rogosa and Sharpe (Merck^TM^, Darmstadt, Germany) broth and agar containing 2% (*w/v*) of monosodium glutamate (Ajinomoto^TM^, Bangkok, Thailand), MRS-MSG, were used as selective media throughout the study. Various kinds of Thai fermented foods including fermented pork (Nham), fermented beef, fermented fish, sausages and fermented vegetables were used for isolation. One gram of each Thai fermented food sample was suspended in 9 mL of sterile normal saline solution (NSS; 0.85% (*w/v*) NaCl) and mixed thoroughly by a vortex mixer prior to a spread plate technique using MRS-MSG agar. Meanwhile, one gram of each part of mulberry tree (leaves, fruits, branches and stems) was enriched in MRS-MSG broth and aerobically incubated at 37 °C for 24 h before the spread plate technique on MRS-MSG agar. All culture plates were aerobically incubated at 37 °C for 24 h. All morphologically different colonies were re-streaked on MRS-MSG agar to obtain pure cultures. All Gram-positive bacteria with negative catalase test were kept at −20 °C until used [16].

### 2.2. Primary Screening of GABA-Producing LAB Using Thin-Layer Chromatography

Each LAB isolate was grown in MRS-MSG broth and aerobically incubated at 37 °C for 48 h. Each culture broth was centrifuged (Hettich MIKRO 220R, Tuttlingen, Germany) at 9744× *g* for 10 min at 4 °C to obtain a supernatant which was further screened for GABA by thin-layer chromatography (TLC) method [17,18]. Briefly, one μL of each sample was spotted on an aluminum sheet silica gel 60 F_254_ (Merck^TM^, Darmstadt, Germany). Analytical standard of γ-Aminobutyric acid (Sigma-Aldrich^TM^, St. Louis, MO, USA) was used as a positive control. A solvent mixture of n-butanol:acetic acid:distilled water (5:3:2) was used [18]. Subsequently, a TLC plate was sprayed with 0.5% (*w/v*) of ninhydrin solution (Merck^TM^, Darmstadt, Germany) and then heated at 60 °C for 30 min. A positive band of GABA appeared in red. The TLC positive samples were further confirmed using a method of high-performance liquid chromatography (HPLC).

### 2.3. GABA Quantitative Analysis by Reversed Phase High-Performance Liquid Chromatography (RP-HPLC)

Quantitative analysis of GABA was performed by a reversed-phase high performance liquid chromatography (RP-HPLC) method as described by Cho et al. [9] with slight modifications. Culture broth of each LAB isolate aerobically grown in MRS-MSG broth at 37 °C for 48 h was centrifuged at 9744× *g* for 10 min at 4 °C. Each culture supernatant was collected and prepared for HPLC analysis as described by Kim and Kim [19] and Ratanaburee et al. [20] with slight modifications. Briefly, 1 mL of supernatant was freeze-dried by a lyophilizer (Labconco, Kansas city, MO, USA) dissolved with 1 mL of a solution mixture of EtOH:DI water:triethylamine (Thermo Fisher Scientific^TM^, Loughborough, UK) (2:2:1) to which was added 80 µL solution of EtOH:DI water:triethylamine:phenylisothiocyanate (PITC) (Sigma-Aldrich^TM^, St. Louis, MO, USA) (7:1:1:1). The sample solution was left at ambient temperature for 20 min and then filtered through 0.45 µm nylon filter (CNW Technologies, Shanghai, China). The HPLC system (1200 series, Agilent Technologies, Inc., Santa Clara, CA, USA) equipped with an ultraviolet (UV) detector was used. GABA content was analyzed by Intersil^®^ ODS-3 column (4.6 × 150 mm, 5 µm) (GL Sciences, Shinjuku-ku, Japan). Mobile phase solution system was comprised of (A); 1.4 mM of sodium acetate (RCI Labscan, Bangkok, Thailand), 0.1% of trimethylamine and 6% of acetonitrile (RCI Labscan), pH 6.1 and (B); 60% acetonitrile. Both mobile phases were filtered through 0.45 µm nylon membrane filter (Filtrex Technologies, Bengaluru, India). The column was eluted for 70 min with a linear gradient of 0–100% at flow rate 1.0 mL/min with mobile phase B [10]. All sample peaks were detected at 254 nm. The authentic GABA standard (Sigma-Aldrich^TM^) was used to set a standard curve and the GABA content of each sample was calculated by the equation: y = 14.782x − 0.0408 (R^2^ = 0.9998).

### 2.4. Genomic DNA Extraction and Amplification of the Selected GABA-Producing LAB

The genomic DNA of the selected bacterial isolate was extracted using the phenol-chloroform extraction method as described by Giraffa et al. [21] with slight modifications. Briefly, the selected bacterial isolate was aerobically grown in MRS-MSG broth at 37 °C for 24 h. To obtain the bacterial cell pellets, 1 mL of culture broth was centrifuged at 9744× *g* for 10 min at 4°C and the supernatant was discarded. Subsequently, mixture of phenol:chloroform:isoamyl alcohol (25:24:1) was added to a sample tube and mixed well prior to centrifugation at 21,924× *g* for 5 min and the supernatant was transferred to a new tube. DNA was precipitated with 3 M of CH_3_COONa (pH 5.2). Isopropanol was added to a sample tube followed by cold absolute ethanol, mixed, incubated at −20 °C for 15 min and then centrifuged at 21,924× *g* for 10 min. Supernatant was removed and the DNA sediment was washed with 70% ethanol and centrifuged at 21,924× *g* for 10 min. DNA was dried for 30 min, dissolved with distilled water and kept at −20 °C.

A polymerase chain reaction (PCR) reaction was carried out in a total volume of 20 μL. The reaction mixture contained 2 μL of 10× reaction buffer (iNtRON Biotechnology, Gyeonggi-do, Korea), 2 μL of 2.5 mM dNTP mixture, 0.2 μL of 50 mM MgCl_2_, 0.5 μL of 100 μM forward primer 27F (5’-AGAGTTTGATCCTGGCTCAG-3´), 0.5 μL of 100 μM reverse primer 1492R (5´-GGTTACCTTGTTACGACTT-3´), 0.3 μL of 5 U/μL *Taq* DNA polymerase, 1 μL of DNA and 13.5 μL of distilled water. The amplification procedures were carried out in a PCR Thermal Cycler (Labcycler, Göttingen, Germany) under the conditions of initial denaturation at 94 °C (2 min), 35 cycles at 94 °C (20 s), 55 °C (20 s), 72 °C (1 min) and final extension at 72 °C (5 min). The PCR product was determined by electrophoresis on 0.8% (*w/v*) agarose gel containing 1× nucleic acid staining solution (RedSafe^®^, iNtRON Biotechnology Inc., Gyeonggi-do, Korea). Gel was detected by UV illumination gel documentation and photographed with a digital camera (GENEFLASH, Cambridge, UK). The PCR product was purified and sequenced by First BASE laboratories Sdn Bhd., Malaysia. Blast analysis was carried out by EzBioCloud (https://www.ezbiocloud.net/) [22] and NCBI database (https://www.ncbi.nlm.nih.gov/) [23], and the phylogenetic tree was constructed by the MEGA7 program [24].

### 2.5. Probiotic Properties and Characteristics of the Selected GABA-Producing LAB

#### 2.5.1. Acid and Bile Salts Tolerance

*L. brevis* F064A was aerobically grown in MRS broth at 37 °C for 24 h. Tolerance to an acidic condition was examined by adjusting MRS broth to pH 2.0 and pH 3.0 with hydrochloric acid [25]. Tolerance to bile salts was examined in MRS broth supplemented with 0.3% (*w/v*) of bile salts (Himedia^®^ Laboratories, Mumbai, India). Initial suspension of *L. brevis* F064A was prepared up to 10^6^–10^8^ colony-forming units (CFU)/mL (OD_625_ = 0.08–0.1) before inoculation [26]. Determination of viable cell count was undertaken every 1 h for 6 h on MRS agar as described by Shekh et al. [25] with slight modifications.

#### 2.5.2. Auto-Aggregation Assay

An auto-aggregation assay was performed following the protocol of Rungsirivanich et al. [27]. *L. brevis* F064A was aerobically grown in MRS broth at 37 °C for 24 h and cell pellets were collected after centrifugation at 3508× *g* 4 °C for 5 min. Cells were resuspened in 1× phosphate-buffered saline (PBS) and adjusted to have turbidity of 0.1 at 600 nm (OD_i_) before standing at ambient temperature. The absorbance of the upper suspension was determined (OD_t_) between 1 and 48 h. The result of auto-aggregation percentage was calculated with the following equation: Auto aggregation (%) = (1 − (OD_t_/OD_i_)) × 100

#### 2.5.3. Cell Surface Hydrophobicity Assay

The hydrocarbon adherence ability of the selected strain was conducted following the protocol of Rungsirivanich et al. [27]. *L. brevis* F064A was aerobically grown in MRS broth at 37 °C for 24 h, cell harvested by centrifugation, washed twice with 1× PBS and resuspended in 1× PBS to obtain the turbidity of OD_600_ = 0.1 (A_initial_). The resuspended cells (5 mL) and xylene (0.5 mL) were added to the test tube and mixed with a vortex mixer. The mixture was left to stand for 30 min at ambient temperature. The bottom aqueous phase was carefully collected and measured for final absorbance (A_final_). Cellular surface hydrophobicity percentage was calculated with the following equation: Cellular surface hydrophobicity (%) = ((A_initial_ − A_final_)/A_initial_) × 100.

#### 2.5.4. In Vitro Bacterial Adhesion Assay

Vero cells were propagated in Dulbecco’s Modified Eagle Medium (DMEM, Gibco^TM^, Paisley, UK) containing 10% (*v/v*) fetal bovine serum (Capricorn Scientific GmbH, Ebsdorfergrund, Germany) and 100 units/mL of penicillin/streptomycin (Caisson Laboratories, Inc., Smithfield, UT, USA) and incubated in CO_2_ incubator at 37 °C for 24 h. This assay was slightly modified from Jacobsen et al. [28]. The harvested Vero cells were plated in 6-well plate with microscopic cover glass approximately 10^3^ cells/well and incubated in CO_2_ incubator at 37 °C for 24 h. Overnight cultures of bacterial cell pellets of *L. brevis* F064A, *E. coli* O157:H7 DMST 12743 and *Shigella dysenteriae* DMST 1511 (as controls) were collected after centrifugation and approximately 10^8^ cells/mL were resuspended in DMEM without antibiotics. Media of each well was removed before inoculation of bacterial suspension (1 mL) and new DMEM (1 mL). Plates were incubated in a CO_2_ incubator for 1 h. Subsequently, each well was washed 3 times with 1× PBS and fixed with methanol for 5 min. After methanol removal, cells were stained with 0.38% Giemsa stain for 15 min. Each experiment was triplicated. Adhesion capacity of bacteria was counted amount of bacterial cells adherent on 100 Vero cells per well under a compound microscope. Percentage of bacterial adhesion was determined.

#### 2.5.5. Blood Haemolysis Activity

MRS agar supplemented with 5% (*v/v*) of human blood was used for *L. brevis* F064A cultivation and aerobically incubated at 37 °C for 48 h. The characteristic appearance around bacterial colony was observed [25].

#### 2.5.6. Antibiotic Susceptibility Assay

*L. brevis* F064A was aerobically grown in MRS broth and turbidity adjusted to obtain cell density equivalent to McFarland standard No. 0.5 before being swabbed on the MRS agar plate. Antibiotic susceptibility test was investigated triplicate using antibiotic discs (HiMedia^®^, Mumbai, India) consisting of co-trimoxazole (25 µg), amoxyclav (30 µg), gentamicin (10 µg), tetracycline (30 µg), ofloxacin (5 µg), cefuroxime (30 µg), amikacin (30 µg), ampicillin (30 µg), ampicillin/sulbactum (10/10 µg), ceftriaxone (30 µg), ticarcillin/clavulanic acid (75/10 µg), cefixime (5 µg), cefotaxime (30 µg), cefoxitin (30 µg) and meropenem (10 µg) according to the method described by Clinical Laboratory Standards Institute (CLSI) [29]. Inhibition zones (mm) were measured.

### 2.6. Mulberry Juice (MJ) Preparation and GABA-Fermented Mulberry Juice (GABA-FMJ) Production

Mulberry fruit (*Morus alba* Linn.) was purchased from farmers in Chiang Mai province, Thailand and was frozen at −20 °C before use. Mulberry fruits was blended and crushed to obtain mulberry juice (MJ). MJ was divided into 2 groups as MJ (control) and MJ supplemented with 2% (*w/v*) of MSG (MJ-MSG). Prior to fermentation, MJ and MJ-MSG were pasteurized at 73 °C for 15 sec before being transferred to sterile Erlenmeyer flasks.

*L. brevis* F064A was grown in MRS broth at 37 °C for 24 h, centrifuged and prepared to 1 × 10^8^ CFU/mL in normal saline solution before inoculation (5% *v/v*) [20]. All cultures were aerobically incubated at 37 °C for 48 h. Samples were collected for analyses.

### 2.7. Analyses of MJ and GABA-FMJ

#### 2.7.1. Reducing Sugar Content, Brix and pH Analyses of MJ

Reducing sugar was determined by the dinitrosalicylic acid (DNS) method using glucose (0–1000 µg/mL) as a standard as described by Miller [30] with slight modifications.

Total soluble solid (°Brix) was measured by a portable refractometer (Trans Instruments, Singapore). The pH was measured by a pH meter (OHAUS^®^, Parsippany, NJ, USA).

#### 2.7.2. Bacterial Growth and pH Determination of GABA-FMJ

During fermentation, viable cells of *L. brevis* F064A were evaluated at 0, 24 and 48 h by the 10-fold serial dilution spread plate technique on MRS agar plates. Change of pH during fermentation was analyzed by a pH meter (OHAUS^®^).

#### 2.7.3. GABA Determination of MJ and GABA-FMJ

GABA content of GABA-fermented mulberry juice (GABA-FMJ) was evaluated by the method mentioned in Section 2.3.

#### 2.7.4. DPPH Scavenging Assay

DPPH (2,2-diphenyl-1-picryl-hydrazyl-hydrate) (Sigma-Aldrich^TM^), 0.1 mM, was prepared in methanol. Gallic acid (Sigma-Aldrich^TM^) solution was dissolved in methanol and used as standard in a range of 0.001–0.01 mg/mL. The standard and samples were reacted with 0.1 mM of DPPH solution. Reaction tubes were incubated in the dark for 20 min and then their absorbance was measured by a spectrophotometer at 517 nm [15]. The half maximal inhibitory concentration of (IC_50_) of standard and sample were calculated. Afterwards, scavenging activity was calculated following the equation:

(1) $$\mathrm{DPPH}\;\mathrm{scavenging}\;\mathrm{activity}\;=\;\frac{{\mathrm{IC}}_{50}\;\mathrm{standard}}{{\mathrm{IC}}_{50}\;\mathrm{sample}}$$

#### 2.7.5. Total Anthocyanin Determination

The pH differential assay was performed for total anthocyanin measurement as described by Hosseinian et al. [31] with slight modifications. Two buffers were prepared, 0.03 M of KCl (pH 1.0) and 0.4 M of sodium acetate trihydrate (pH 4.5). Twenty µL of each sample were added into 180 µL of KCl buffer and 20 µL of sample were added into 180 µL of sodium acetate buffer in different well of 96-well plate. Each sample was analyzed in triplicate. Afterwards, the mixture sample was incubated at ambient temperature for 15 min before absorbance measurement at 510 nm and 700 nm. The concentration of anthocyanin was calculated and reported as Cyanidin-3-glucoside equivalents following the formula:

$$\mathrm{Total}\;\mathrm{Anthocyanin}\;=\;\frac{A\times \mathrm{MW}\times \mathrm{DF}\times 10^{3}}{\varepsilon \times 1}$$

* *A* is absorbance = (A_510_ – A_700_) pH 1.0 – (A_510_ – A_700_) pH 4.5, MW (g/mol) of Cy-3-glc is 449.2, DF is dilution factor of sample, ε is the extinction coefficient of Cy-3-glc (26,900 *L* × cm^−1^ × mol^−1^) where *L* is 1 (pathlength in cm).

#### 2.7.6. Antibacterial Activity Assay

The agar well diffusion method was performed for antibacterial activity of MJ and GABA-FMJ. *Staphylococcus aureus* ATCC 25923, *Escherichia coli* ATCC 25922, *E. coli* O157:H7 DMST 12743, *Bacillus cereus* TISTR 687, *Salmonella enterica* subspecies *enterica* serovar Typhi DMST 22842, *Shigella dysenteriae* DMST 1511 and *Vibrio cholerae* DMST 2873 were aerobically grown in Mueller Hinton broth (MHB) at 37 °C for 24 h. The turbidity of each culture broth was adjusted to McFarland standard No. 0.5 and swabbed on Mueller Hinton agar plates. One-hundred μL of each sample were dropped into a hole prepared by a cork borer (diameter of 9 mm) and then aerobically incubated at 37 °C for 24 h. Gentamicin (0.1 mg/mL) was used as a positive control. After incubation, the inhibition zone (mm) was observed [32].

#### 2.7.7. Lipid Peroxidation Inhibitory Activity Assay

A thiobarbituric acid reactive substance (TBARs) assay described by Tarladgis et al. 1960 [33], modified by Djeri and Williams [34] and Semeniuc et al. [35] was used to measure the lipid peroxidation inhibitory activity of *L. brevis* F064A in unfermented MJ and GABA-FMJ. Malondialdehyde (MDA) solution (0–100 µM) was presented as standard. For standard, 200 µL of each concentration of MDA solution were added with 200 µL of 0.67% (*w/v*) thiobarbituric acid (TBA) and boiled for 10 min. For sample, 200 µL of sample were mixed well with 400 µL of 10% (*w/v*) trichloroacetic acid (TCA) and centrifuged at 9,744 × *g* 4 °C for 15 min. Afterwards, 400 µL of supernatant was collected and mixed with 0.67% TBA and boiled for 10 min. After being cooled down at room temperature, mixture solution absorbance was measured at 532 nm. The lipid peroxidation inhibitory activity of sample was calculated following the equation of the standard curve: y = 0.0222x + 0.0473 (R^2^ = 0.9987).

## 3. Results

### 3.1. Bacterial Isolation, Screening and 16S rRNA Gene Identification of GABA-Producing LAB

**Figure 1:**
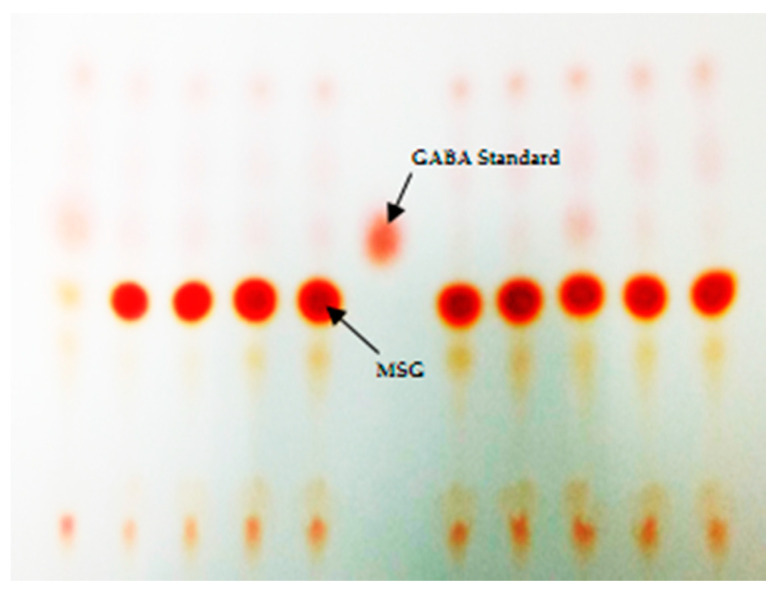
Screening of γ-aminobutyric acid (GABA)-producing lactic acid bacteria (LAB) by the thin-layer chromatography (TLC) method after reacted with ninhydrin solution and heated at 60 °C for 30 min.

We isolated 127 isolates of LAB from some Thai fermented foods and parts of the mulberry tree including fermented pork (63 isolates), fermented beef (8 isolates), fermented fish (14 isolates), fermented vegetables (23 isolates), fermented sausages (14 isolates), mulberry leaves (3 isolates) and mulberry fruits (2 isolates). All of them showed Gram-positive and negative catalase tests. After screening by the TLC method, 36 isolates showed the red spot which matched the GABA standard (Figure 1) and they were selected for the confirmation step by the HPLC method.

**Figure 2:**
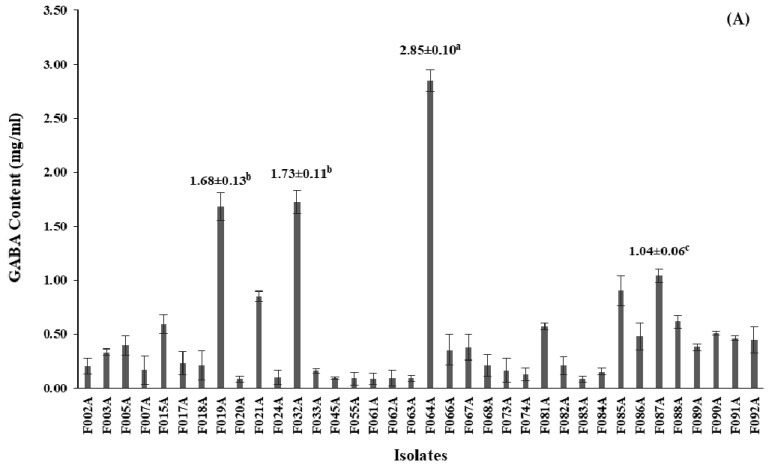

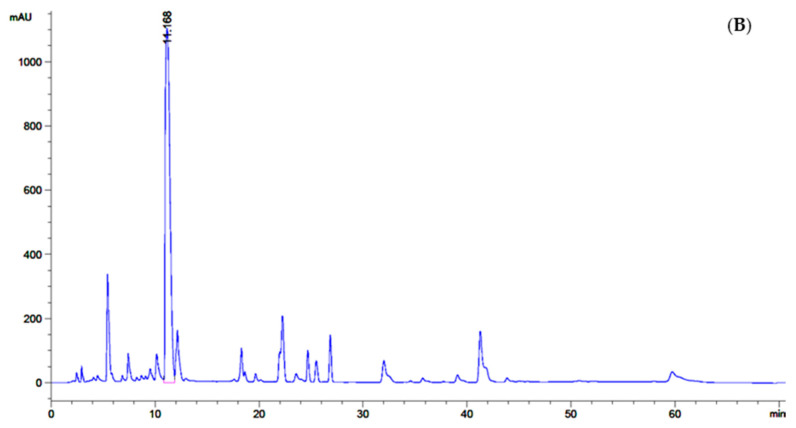
Screening of GABA-producing LAB; (**A**) GABA content (mg/mL) from 36 isolates after 24 h of cultivation in De Man, Rogosa and Sharpe (MRS-MSG) broth. The bars represented mean ± standard deviation (S.D., *n* = 2); (**B**) chromatogram showed peak area (mAU) of GABA content (retention time at 11.168) detected by high-performance liquid chromatography (HPLC) after being derivatized with phenylisothiocyanate (PITC) solution. The statistical analysis was performed by using one-way analysis of variance (ANOVA) Values with different superscript letter in Figure 2A are considered statistically significant difference at *p* < 0.05.

For quantitative analysis of the selected GABA-producing LAB performed by HPLC, GABA contents of all 36 isolates were produced in the range of 0.08–2.85 mg/mL (Table S1). Among 36 isolates, 4 isolates designed as F019A, F032A, F064A and F087A had GABA content higher than 1.00 mg/mL, however, the isolated F064A significantly (*p ≤* 0.05) produced higher GABA content at 2.85 ± 0.10 mg/mL than the other isolates. GABA contents of all isolates are shown in Figure 2.

**Figure 3:**
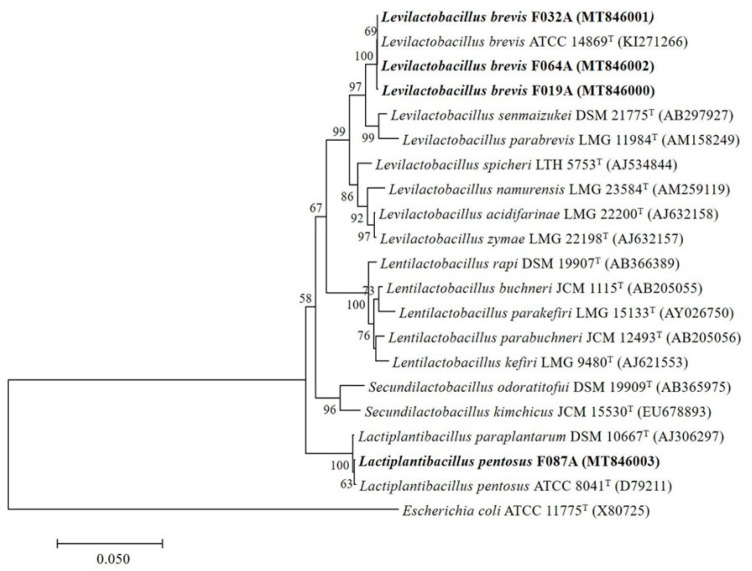
Phylogenetic relationships of 4 selected GABA-producing LAB isolates including F019A, F032A, F064A and F087A (bold), comparison with some species of the lactic acid bacteria based on the sequence of 16S rRNA gene using universal primer 27F and 1492R. The branching pattern was generated using the neighbor-joining method by MEGA7 software. Bootstrap values; expressed as percentage of 1000 replications, more than 50% are shown at the branch points. Scale bar, 0.050 represented substitution per nucleotide position. *Escherichia coli* ATCC 11775^T^ (X80725) is presented as out group sequence.

The isolate F064A which was isolated from Thai fermented sausage (Sai Krok Isan) was identified by 16S rRNA gene sequencing analysis with a length of 1427 bp by the BLAST search program. The 16S rRNA gene sequence of the isolate F064A showed 100% sequence similarity and close to the strain *Levilactobacillus brevis* KI271266 (Figure 3). Therefore, the strain F064A was *Levilactobacillus brevis* F064A (MT846002).

The 16S rRNA gene sequence of the isolate F032A isolated from fermented pork purchased from Lampang province and the isolate F019A isolated from small fermented pork obtained from Chiang Mai province displayed 99.72% sequence similarity to *Levilactobacillus brevis* KI271266 (Figure 3). Therefore, the strains F032A and F019A were identified as *Levilactobacillus brevis* F032A (MT846001) and *Levilactobacillus brevis* F019A (MT846000), respectively. The isolate F087A isolated from fermented meat purchased from Chiang Rai province had 99.79% sequence similarity to *Lactiplantibacillus pentosus* ATCC 8041T (D79211) and was named *Lactiplantibacillus pentosus* F087A (MT846003).

### 3.2. Probiotic Properties

#### 3.2.1. Acid and Bile Salts Tolerance

**Figure 4:**
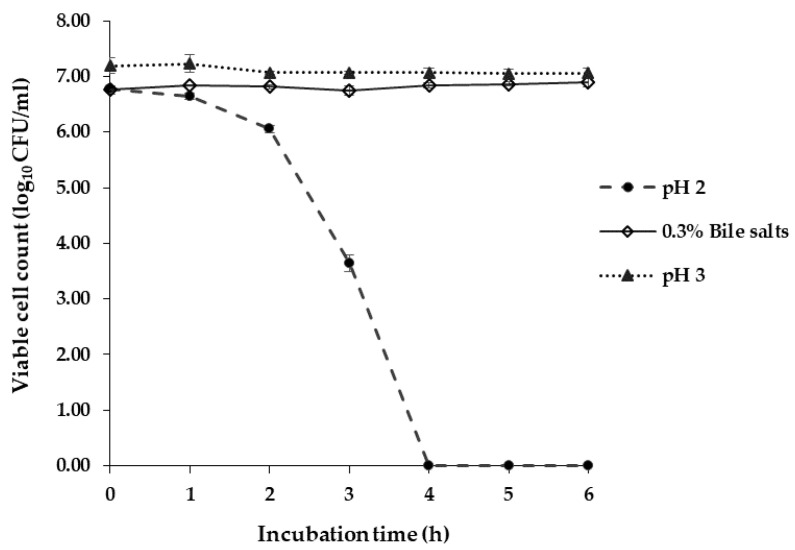
Viable cell count (log_10_ colony-forming units (CFU)/mL) of *L. brevis* F064A against acidic condition (pH 2.0 and pH 3.0) and 0.3% (*w/v*) of bile salts. The experiments were triplicate. The results represented as mean ± S.D. (*n* = 3).

At pH 2.0, *L. brevis* F064A was able to survive after 3 h of incubation with viable cells decreased from 6.78 ± 0.04 (0 h) to 3.64 ± 0.15 log CFU/mL (3 h). They could not survive at pH 2.0 after 4 h of incubation. At pH 3.0, the bacteria survived more than 6 h of incubation and the viable cell count was rather stable from 7.19 ± 0.14 (0 h) to 7.0 6± 0.09 log CFU/mL (6 h). *L. brevis* F064A survived in 0.3% (*w/v*) of bile salts more than 6 h of incubation which showed a quantity of viable cells from 6.76 ± 0.08 (0 h) to 6.90 ± 0.08 log CFU/mL (6 h) (Figure 4).

#### 3.2.2. Auto-Aggregation and Surface Hydrophobicity

**Figure 5:**
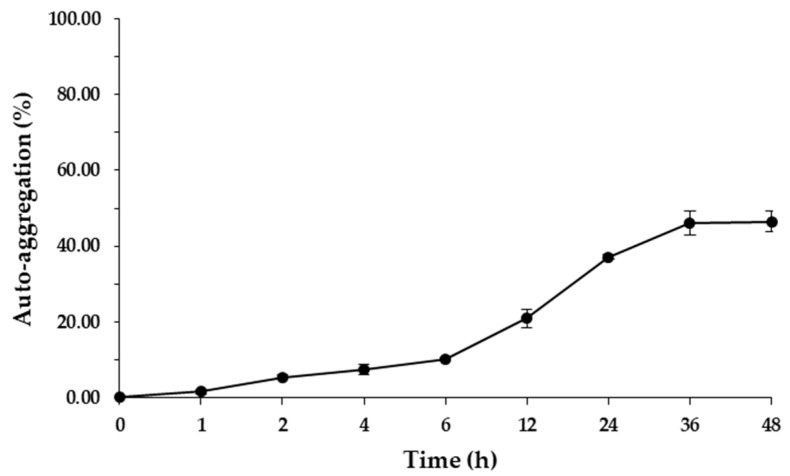
Percentage of auto aggregation ability of *L. brevis* F064A during 48 h of incubation. The results represented as mean ± S.D. (*n* = 3).

*L. brevis* F064A showed the percentage of auto aggregation between 1.52 ± 0.56% (at 1 h of incubation) to 46.46 ± 2.65% after 48 h of incubation (Figure 5). *L. brevis* F064A displayed low cellular surface hydrophobicity of 2.22 ± 0.48% at 30 min of incubation.

#### 3.2.3. Bacterial Adhesion and Blood Haemolysis

**Figure 6:**
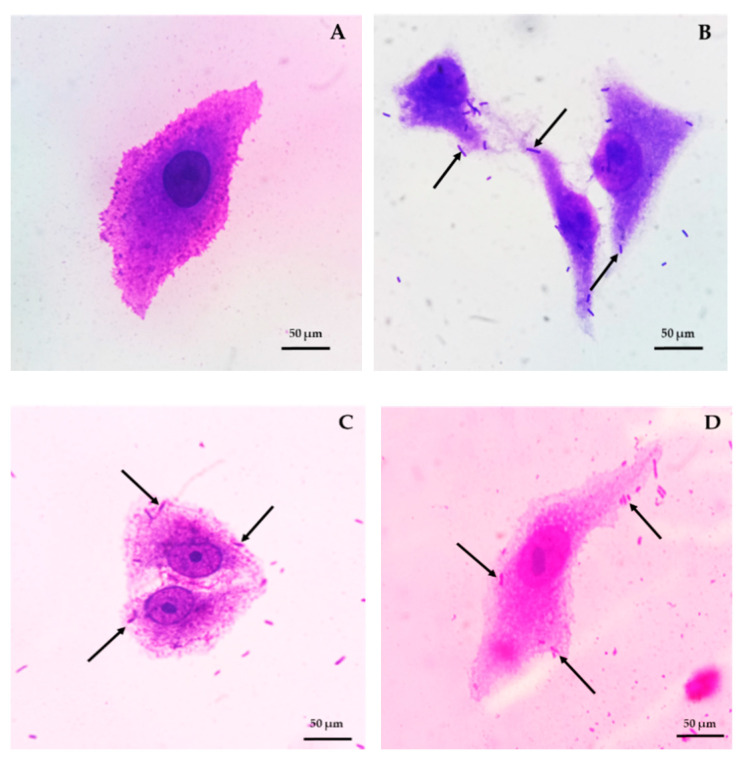
Vero cell control (**A**). Bacterial adeshion of *L. brevis* F064A (**B**), *E. coli* O157:H7 DMST 12743 (**C**) and *Shigella dysenteriae* DMST 1511 (**D**).

*L. brevis* F064A presented the highest adhesion ability on Vero cells as 171.8% and 100% when *Shigella dysenteriae* DMST 1511 and *E. coli* O157:H7 DMST 12743 were used as controls, respectively (Figure 6).

*L. brevis* F064A strain showed no haemolytic activity and was considered as γ-haemolysis type.

#### 3.2.4. Antibiotic Susceptibility

*L. brevis* F064A was susceptible to 14 from 15 testing antibiotics with inhibition zone ranging between 6.3 to 42.0 mm and resistant to Ticarcillin/Clavulanic acid. Inhibitory clear zone of antibiotics were co-trimoxazole (26.7 ± 1.5 mm), amoxyclav (33.0 ± 1.0 mm), gentamicin (13.0 ± 3.5 mm), tetracycline (25.7 ± 1.5 mm), ofloxacin (10.3 ± 2.3 mm), cefuroxime (31.0 ± 1.7 mm), amikacin (18.7 ± 1.2 mm), ampicillin (32.7 ± 2.3 mm), ampicillin/sulbactum (29.7 ± 4.5 mm), ceftriaxone (31.0 ± 2.6 mm), cefixime (18.0 ± 2.6 mm), cefotaxime (40.7 ± 1.2 mm), cefoxitin (6.3 ± 1.5 mm) and meropenem (42.0 ± 2.0 mm) (Figure S1).

### 3.3. Characteristics of MJ and GABA-FMJ

#### 3.3.1. Reducing Sugar, Total Soluble Solid, GABA content and pH of MJ

**Figure 7:**
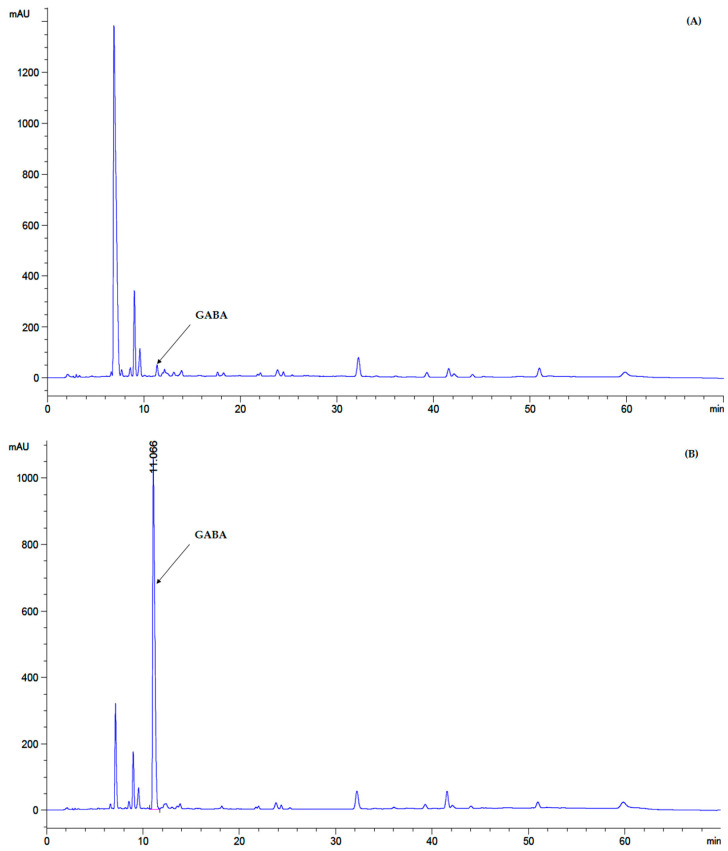
Chromatogram of GABA analysis of GABA-FMJ at 0 h (**A**) and 48 h (**B**) of incubation.

Initial parameters of MJ were indicated. Reducing sugar was 46.50 ± 2.35 mg/mL. Total soluble solid (sugar content of sugar solution) was 8.2 °Brix. GABA content was less than 0.10 ± 0.02 mg/mL (Figure 7A), and initial pH value without any additives was 3.64 ± 0.42.

#### 3.3.2. Bacterial Growth, pH and GABA Content of GABA-FMJ

After inoculation, viable cell count was exponentially increased from 6.68 ± 0.09 (0 h) to 10.54 ± 0.45 log_10_ CFU/mL at 48 h of incubation and decreased to 8.91 ± 0.31 log_10_ CFU/mL after 72 h of incubation. Moreover, pH was changed from 3.88 ± 0.01 (0 h) to 4.12 ± 0.02 after 48 h of incubation.

Additionally, in GABA-FMJ the GABA content increased from 0.1 mg/mL (control) to 3.31 ± 0.06 mg/mL (*p* ≤ 0.05) (Figure 7B).

#### 3.3.3. DPPH Scavenging Activity and Total Anthocyanin of GABA-FMJ

DPPH scavenging activity of MJ was 4.41 ± 0.02 mg gallic acid equivalent (GAE)/mL while GABA-FMJ was 5.58 ± 0.52 mg GAE/mL (increased by 26.53%) after 48 h of incubation (*p ≥* 0.05). Additionally, total anthocyanin content of GABA-FMJ was insignificantly decreased by 10.83% from 263.18 ± 24.82 to 234.68 ± 15.53 mg cyanidin-3-glucoside/mL.

#### 3.3.4. Antibacterial Ability of MJ and GABA-FMJ

MJ was unable to inhibit growth of test enteric pathogenic bacteria while GABA-FMJ was able to inhibit growth of *B. cereus* TISTR 687, *Salmonella* Typhi DMST 22842 and *Shi. dysenteriae* DMST 1511 with the inhibition zone diameter of 13.58 ± 1.26, 20.83 ± 0.76 and 16.56 ± 4.07 mm, respectively.

#### 3.3.5. Lipid Peroxidation Inhibitory Activity of GABA-FMJ

After fermentation, GABA-FMJ showed lipid peroxidation inhibitory activity as 0.43 ± 0.23 µg MDA/mL while unfermented MJ showed 1.14 ± 0.27 µg MDA/mL. The percentage inhibition of MDA in GABA-FMJ was 62.28%.

## 4. Discussion

Most sources of GABA-producing LAB isolation are fermented foods or other glutamic acid-consisting sources. In this study, the highest capacity of GABA-producing LAB, *L. brevis* F064A, was isolated from Thai fermented sausage (*Sai Krok Isan*). Previous reports have shown many GABA-producing LAB species from different sources such as *Lactiplantibacillus plantarum* L10-11 was isolated from *plaa-som* (Thai fermented fish) [36], *Lactiplantibacillus pentosus* SS6 was isolated from fermented mulberry fruit [37], *Companilactobacillus futsaii* CS3 was isolated from Thai fermented shrimp or *Kung-Som* [38], *Latilactobacillus sakei* A156 was isolated from Korean fermented seafood or *Jeot-gal* [39], three strains of *L. brevis* and one of *Lactococcus lactis* were isolated from Spanish cheese [40], five strains including *Lactiplantibacillus plantarum, L. brevis*, *Leuconostoc mesenteroides*, *Leuconostoc lactis* and *Weissella viridescens* were isolated from kimchi [19], *Companilactobacillus farciminis* D323 was isolated from a Myanmar traditional fermented fishery product [17], *Lacticaseibacillus paracasei* NFRI 7415 was isolated from Japanese traditional fermented fish or *Funa-sushi* [41]. In addition to fermented foods, GABA-producing LAB as *L. plantarum* Taj-Apis362 was isolated from honeybees [42]. Some reports also reveal that *L. brevis* DSM 32386 can produce 0.262 mg/mL of GABA [43] while *L. brevis* GABA100 produces 27.6 mg/mL of GABA [44]. The GABA-producing capacity of each LAB strain is dependent on glutamate decarboxylase enzyme and many factors.

Nowadays, most probiotic bacteria are classified to the genera *Lactobacillus*, *Bifidobacterium*, and include some species of the genera *Lactococccus*, *Enterococcus* and *Saccharomyces* [45]. In this study, *L. brevis* F064A could tolerate pH 2.0, pH 3.0 and 0.3% bile salts; according to many previous studies such as *L. paracasei* K5 can tolerate pH 2.0–4.0 at 2 h incubation [46] and *C. futsaii* can tolerate simulated stomach condition at pH 2.5 for 2 h [47]. Normally, pH value in the human stomach ranges between 1.0–2.0, but can be up to 3.0 or higher with the presence of food. The average concentration of bile salts in humans is 0.3% and food digestion process takes approximately 2–4 h [25,26]. Tolerance to bile salts of *Lactobacillus* strains is relied on its capability to hydrolyze bile salts by specific enzymes such as bile salt hydrolase (BSH) [26]. *L. brevis* F064A presented auto aggregation ability less than 50% after 48 h of incubation. High auto-aggregation ability which is strain-specific can provide probiotic bacteria to persist in the intestinal tract and associate with adherence to mucus and epithelial cells. However, some studies have reported that a high hydrophobicity level leads to higher capacity to adhere on Caco-2 cells [48,49]. On the other hand, some research has suggested that lower percentages of auto aggregation can prevent probiotic bacteria from biofilm formation of enteric pathogenic bacteria [48,49]. *L. brevis* F064A showed a lower hydrophobicity level. The hydrophobicity property indicates the adhesion capacity on epithelial cells of probiotic bacteria but it does not confirm that a high level of hydrophobicity can show a strong adhesion to mucosa or epithelial cells [50,51]. Additionally, the hydrophobicity ability had a high variation even in the same strain of bacteria such as 7 strains of *L. fermentum* present a variation of hydrophobicity in a range from 0.30–68.81% [51]. Blood haemolysis is one of safety properties for LAB. In this study, *L. brevis* F064A was unable to damage red blood cells. Normally, blood haemolysis and DNase capacity are presented as virulence factors for pathogenic microorganisms [25]. Antibiotic susceptibility is a criterion of safety attribute evaluation for probiotic bacteria. *L. brevis* F064A was susceptible to all testing antibiotics but resistant to ticarcillin/clavulanic acid which is a type of penicillin antibiotic. According to Gad et al. [52], lactic acid bacteria is highly susceptible to many beta-lactam antibiotics except penicillin. Ticarcillin is penicillin antibiotic combined with clavulanic acid (beta-lactamase inhibitor) which is generally used against Gram-negative bacilli and enterococci [53].

From the previous study, mulberry juice was individually fermented with 3 strains of *Lactobacillus* including *L. plantarum*, *L. acidophilus* and *L. paracasei* which improve their antioxidant attributes but there is no study about GABA [54]. Furthermore, mulberry juice has been fermented with a single culture of *Saccharomyces cerevisiae* SC125 which produced 1.45 g/L of GABAor *L. plantarum* BC114 which produced 1.03 g/L of GABA while a co-culture of starters could increase GABA content to 2.42 g/L [55]. In this study, mulberry juice with 2% of MSG was fermented by *L. brevis* F064A. The results indicated that GABA-FMJ could increase bacterial growth during fermentation, GABA content, and antioxidant and antibacterial activities while total anthocyanin was decreased (non-significantly) and also showed lipid peroxidation inhibitory. The fermentation of mulberry with LAB are expected to increase many functional compounds [56]. GABA may be increased due to acidic condition of mulberry juice with glutamate which is a substrate to produce GABA via the GAD system to maintain the cytosolic pH of cells. A study of fermented sweet lemon juice by *L. plantarum* LS5 presents that the cell count is increased from 7.01 ± 0.01 to 8.63 ± 0.38 log CFU/mL at 37 °C for 48 h, the antibacterial activity is improved, the antioxidant activity is increased but the phenolic compound is decreased [57]. Increasing antioxidant activity was agreed with Mousavi et al. [58] who studied fermentation of pomegranate juice fermented with *L. plantarum* and *Lactobacillus acidophilus*. Sung et al. [59] shows that antioxidant activity of yogurt supplemented with freeze-dried mulberry increased with no significant level during storage. In this study, increasing antioxidant activity (DPPH assay) and decreasing anthocyanin (Cy-3-gluc) were related to Mousavi et al. [58]. Increasing antioxidant activity is due to the hydrolytic enzyme produced by LAB which can hydrolyze complex phytochemicals to simple structure [54]. Anthocyanin stability during fermentation depends on many factors as type of anthocyanin, light, temperature, pH and enzymes including oxygen. Moreover, hydroxyl and methoxyl groups can decrease anthocyanin stability including that metabolized by a LAB starter [58,60]. The GABA-FMJ product can inhibit growth of some enteric pathogenic bacteria both Gram-positive (*B. cereus*) and Gram-negative (*Salmonella* Typhi *and Shigella dysenteriae*) bacteria due to a mixture of some organic acids, low pH, diacetyl compound and some unknown or unidentified compounds from extracellular substances by LAB [61]. Growth inhibition of Gram-positive bacteria was less sensitive than Gram-negative bacteria. However, Kantachote et al. [61] reports that some fermented beverage plants (FBPs) can inhibit growth of *B. cereus* and *S. aureus*. In addition, lactic acid can also damage the lipopolysaccharides of the outer membrane of Gram negative bacteria [62]. Malondialdehyde (MDA) is a product of lipid damage [35]. This study suggests that *L. brevis* F064A can be promoted to increase the value of fermented mulberry juice and will lead to functional food product development.

## 5. Conclusions

*L. brevis* F064A, GABA-producing lactic acid bacteria, isolated from Thai fermented food was considered as a potential human probiotic by its tolerance to acidic condition and bile salt and its ability to adhere to Vero and mucosal cells. Additionally, *L. brevis* F064A could apply to the fermentation product to improve their bioactive compounds. GABA-FMJ fermented by *L. brevis* F064A presented an ability to enhance the growth of probiotics, increase GABA content, improve antibacterial and antioxidant activities, and had lipid peroxidation inhibitory activity. GABA-FMJ should be further developed to become a potential functional food product containing high GABA and bioactive compounds for good health of people of all ages.

## Data Availability

All data underlying the results are included as part of the published article and its Supplementary Materials.

## Supplementary Materials

The following are available online at https://www.mdpi.com/2076-2607/9/1/33/s1, Table S1: Isolation sources, basic morphology and GABA content (mg/mL) of 36 selected GABA-producing LABs, Figure S1: Inhibition zone (mm) of antibiotic susceptibility test of *L. brevis* F06A with various antibiotic disc group Hexa G-minus 3 (A), Hexa G-minus 11 (B) and Hexa G-minus 19 (C).
