# Supplementary material for: Screening of GABA-Producing Lactic Acid Bacteria from Thai Fermented Foods and Probiotic Potential of Levilactobacillus brevis F064A for GABA-Fermented Mulberry Juice Production
Source: Microorganisms. 2020 Dec 24;9(1):33. doi: 10.3390/microorganisms9010033 (PMC7823765; doi:10.3390/microorganisms9010033)
Supplement: Supplementary file 1 [file microorganisms-09-00033-s001.pdf]

Supplementary Material

Screening of GABA-Producing Lactic Acid Bacteria from Thai Fermented Foods and Probiotic Potential of *Levilactobacillus Brevis* F064A for GABA-Fermented Mulberry Juice Production

Jirapat Kanklai [1,2], Tasneem Somwong [3], Patthanasak Rungsirivanich [1,2] and Narumol Thongwai [1,4,*]

Table S1. Isolation sources, basic morphology and GABA content (mg/ml) of 36 selected GABA-producing LABs.

Code	Source, Province	Gram stain	Cell shape	GABA content (mg/ml) (mean ± SD)
F002A	Fermented pork, Chiang Mai	+	rod	0.20 ± 0.07
F003A	Fermented pork, Chiang Mai	+	rod	0.33 ± 0.02
F005A	Fermented pork, Chiang Mai	+	rod	0.40 ± 0.08
F007A	Fermented pork, Chiang Mai	+	rod	0.17 ± 0.13
F015A	Small fermented pork, Chiang Mai	+	rod	0.59 ± 0.07
F017A	Small fermented pork, Chiang Mai	+	rod	0.23 ± 0.08
F018A	Small fermented pork, Chiang Mai	+	rod	0.21 ± 0.13
F019A	Small fermented pork, Chiang Mai	+	rod	1.68 ± 0.13
F020A	Small fermented pork, Chiang Mai	+	rod	0.08 ± 0.01
F021A	Small fermented pork, Chiang Mai	+	rod	0.85 ± 0.03
F024A	Thai fermented sausage, Chiang Mai	+	rod	0.10 ± 0.04
F032A	Fermented pork, Lampang	+	rod	1.73 ± 0.11
F033A	Fermented pork, Lampang	+	rod	0.16 ± 0.01
F045A	Fermented vegetable, Chiang Mai	+	Cocci	0.09 ± 0.01
F055A	Fermented pork rip, Lampang	+	cocci	0.09 ± 0.03
F059A	Fermented pork rip, Lampang	+	rod	0.09 ± 0.04
F061A	Fermented pork rip, Lampang	+	cocci	0.09 ± 0.03
F062A	Thai fermented sausage, Chiang Mai	+	rod	0.09 ± 0.04
F063A	Thai fermented sausage, Chiang Mai	+	rod	0.06 ± 0.02
F064A	Thai fermented sausage, Chiang Mai	+	rod	2.85 ± 0.10

F066A	Thai fermented sausage, Chiang Mai	+	rod	0.36 ± 0.12
F067A	Thai fermented sausage, Chiang Mai	+	rod	0.38 ± 0.11
F068A	Fermented pork, Chiang Mai	+	rod	0.21 ± 0.11
F073A	Fermented pork, Chiang Mai	+	rod	0.16 ± 0.11
F074A	Fermented Fish, Chiang Mai	+	rod	0.13 ± 0.04
F081A	Fermented fish, Chiang Rai	+	rod	0.57 ± 0.02
F082A	Fermented fish, Chiang Rai	+	rod	0.21 ± 0.08
F083A	Fermented fish, Chiang Rai	+	rod	0.08 ± 0.02
F084A	Fermented fish, Chiang Rai	+	rod	0.15 ± 0.02
F085A	Fermented fish, Chiang Rai	+	rod	0.90 ± 0.13
F086A	Fermented meat, Chiang Rai	+	rod	0.48 ± 0.12
F087A	Fermented meat, Chiang Rai	+	rod	1.04 ± 0.06
F088A	Fermented meat, Chiang Rai	+	rod	0.62 ± 0.04
F089A	Fermented meat, Chiang Rai	+	rod	0.38 ± 0.02
F091A	Fermented meat, Chiang Rai	+	rod	0.51 ± 0.02
F092A	Fermented meat, Chiang Rai	+	rod	0.46 ± 0.12

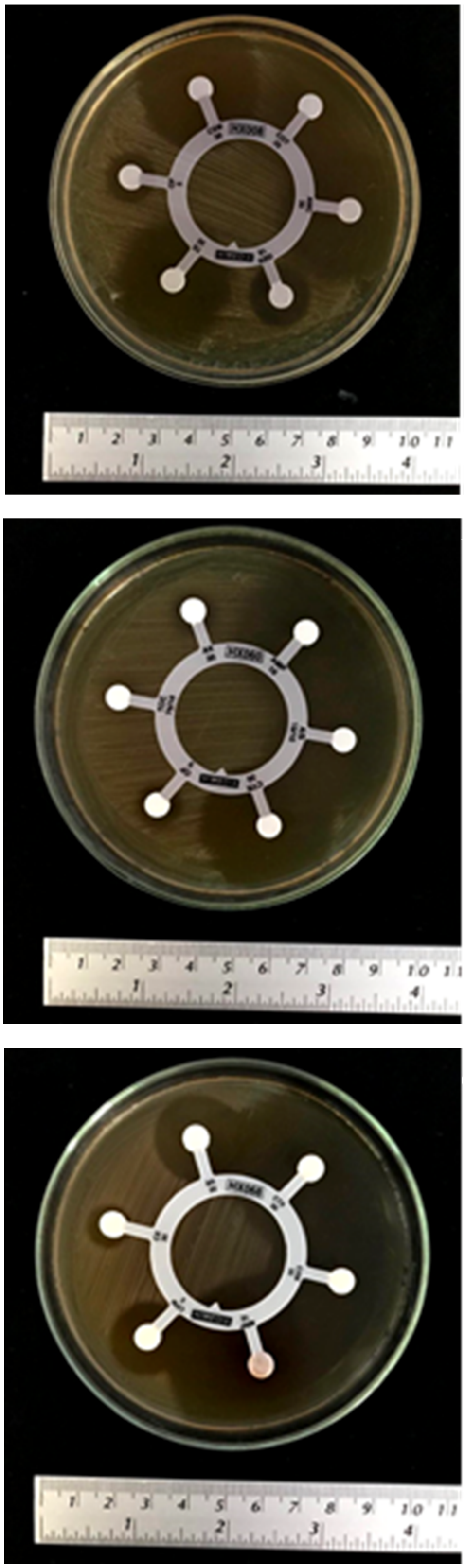

Figure S1. Inhibition zone (mm) of antibiotic susceptibility test of *L. brevis* F06A with antibiotic disc group Hexa G-minus 3 (A), Hexa G-minus 11 (B) and Hexa G-minus 19 (C).
